# Simply Fabricated Inexpensive Dual-Polymer-Coated Fabry-Perot Interferometer-Based Temperature Sensors with High Sensitivity

**DOI:** 10.3390/s21227632

**Published:** 2021-11-17

**Authors:** Tejaswi Tanaji Salunkhe, Ho Kyung Lee, Hyung Wook Choi, Sang Joon Park, Il Tae Kim

**Affiliations:** 1 Department of Chemical and Biological Engineering, Gachon University, Seongnam-si 13120, Korea; tejaswisalunkhe235@gmail.com (T.T.S.); ghrud0722@gmail.com (H.K.L.); psj@gachon.ac.kr (S.J.P.); 2 Department of Electrical Engineering, Gachon University, Seongnam-si 13120, Korea; chw@gachon.ac.kr

**Keywords:** dual-polymer-coated sensor, high sensitivity, Fabry–Perot interferometer, thermosensitive polymer

## Abstract

We designed simply fabricated, highly sensitive, and cost-effective dual-polymer-coated Fabry–Perot interferometer (DFPI)-based temperature sensors by employing thermosensitive polymers and non-thermosensitive polymers, as well as different two successive dip-coating techniques (stepwise dip coating and polymer mixture coating). Seven sensors were fabricated using different polymer combinations for performance optimization. The experiments demonstrated that the stepwise dip-coated dual thermosensitive polymer sensors exhibited the highest sensitivity (2142.5 pm °C^−1^ for poly(methyl methacrylate)-polycarbonate (PMMA_PC) and 785.5 pm °C^−1^ for poly(methyl methacrylate)- polystyrene (PMMA_PS)). Conversely, the polymer-mixture-coated sensors yielded low sensitivities (339.5 pm °C^−1^ for the poly(methyl methacrylate)-polycarbonate mixture (PMMA_PC mixture) and 233.5 pm °C^−1^ for the poly(methyl methacrylate)-polystyrene mixture (PMMA_PS mixture). Thus, the coating method, polymer selection, and thin air-bubble-free coating are crucial for high-sensitivity DFPI-based sensors. Furthermore, the DFPI-based sensors yielded stable readouts, based on three measurements. Our comprehensive results confirm the effectiveness, reproducibility, stability, fast response, feasibility, and accuracy of temperature measurements using the proposed sensors. The excellent performance and simplicity of our proposed sensors are promising for biomedical, biochemical, and physical applications.

## 1. Introduction

Optic fiber-based sensors are becoming increasingly mature and have seen an increasing demand in the fields of biotechnology, energy, superconducting magnets, biomedicine, healthcare, aerospace, automotive technology, and civil engineering [1,2,3]. Over the last few decades, optic fiber-based sensors have attracted increasing attention owing to their potential applicability for temperature [4,5,6], refractive index (RI) [7,8], strain [9], humidity [10,11], pressure [12], and bending [13] measurements. Fiber Bragg gratings (FBG) are the most popular optical fibers that have been utilized in the field of optical fiber temperature sensors for high sensitivity measurements [14,15]. Among the several existing types of fiber-optic sensors, Fabry–Perot interferometer-based temperature sensors have been studied extensively, owing to their advantages over conventional sensors, including durability against unbearable environments, good repeatability, light weight, compact size, excellent accuracy, feasibility, fast response, good sensitivity, wide dynamic range, insensitivity to electromagnetic interference, low cost, high resolution, simple fabrication, and remote sensing ability [16,17,18].

Fabry–Perot interferometer-based temperature sensing utilizes the simple Fresnel reflection principle. This principle accounts for the interference phenomenon owing to the difference between the thermo-optic coefficient (TOC) and the thermal expansion coefficient (TEC) of the coating material. Numerous hybrid intrinsic Fabry–Perot interferometer-based temperature sensors have been developed by combining hollow core fibers, multimode fibers, photonic crystal fibers, and dual-core photonic crystal fibers with single-mode fibers (SMFs) [2,19,20,21]. Such hybrid sensors are highly sensitive; however, they have some disadvantages, such as low reproducibility, high cost, and complicated fabrication. Simple Fabry–Perot interferometer-based temperature sensors were established by coating polymers [1,22,23,24], agarose [25,26], carbon nanotubes [27], porous silica xerogels [28], SU-8 photoresistors [29], UV-curable resins [30], metal oxides, and metal alloys [31] with inexpensive and specific cavity dimensions [32]. However, the crucial disadvantage of such sensors is their low sensitivity, which is restricted by the elastic modulus of the coating material. In order to achieve excellent temperature sensitivity, the Fabry–Perot interferometer structure should be modified by considering the cost, simplicity, and reproducibility, which is challenging and demanding.

With the above motivation, in this article, we describe the design, fabrication, and experimental characterization of low-cost and simple, high-sensitivity dual-polymer-coated Fabry–Perot interferometer (DFPI)-based temperature sensors. The fiber tip of a simple SMF was coated with two thermosensitive polymers by stepwise dip coating, followed by oven drying. For comparison, one thermosensitive polymer and one non-thermosensitive polymer were coated using the same method. On the other hand, a mixture of two polymers was coated as well, and the corresponding temperature measurement results were compared. The fabricated sensors were analyzed using microscopic images and interference patterns. The operation principle of a dual-polymer-coated sensor utilizes the interference of three reflective surfaces. The dual-polymer microcavities create a large optical path and a large change in RI responsible for the high wavelength shift, ensuring high temperature sensitivity. Seven devices, using different polymer combinations and fabricated using different coating methods, were considered, and our experimental results demonstrate that the temperature measurement data for all of the considered sensors were well fitted by polynomials. All of the studied DFPI-based sensors were highly stable and generated reproducible data, as confirmed by three replicate measurements and wavelength fluctuation results with very low standard deviations in terms of the temperature and sensitivity. The stepwise dip-coated PMMA_PC sensor exhibited the best performance (2142.5 pm °C^−1^) for temperatures in the 24.4–80 °C range, which was ascribed to its excellent thermosensitive properties and uniform thin coating. This suggests that the stepwise dip-coating method is promising for obtaining high-quality sensors.

## 2. Materials and Methods

### 2.1. Principle of Operation

**Scheme 1:**
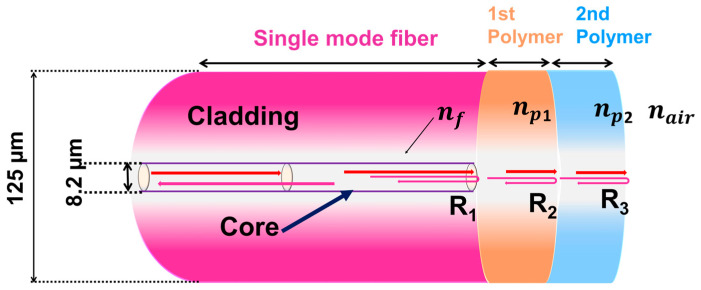
Proposed DFPI-based temperature sensor.

The proposed DFPI-based sensor is represented in Scheme 1, in which a single-mode optic fiber has the RI of n_SMF_ = 1.456 [33]. The tip of the SMF was coated by two polymers with the refractive indices of n_1_ (first polymer) and n_2_ (second polymer). The DFPI-based sensor utilizes the principle of Fresnel reflection. Fresnel’s reflection is an optical phenomenon occurring at the interface between two media with different refractive indices [34,35,36]. As illustrated in Scheme 1, the DFPI-based sensor has a total of three reflection surfaces on the tip, corresponding to the interface between the fiber and the first polymer, the interface between the first and second polymers, and the interface between the second polymer and air; these correspond, respectively, to the Fresnel reflection coefficients R_1_, R_2_, and R_3_. The three reflected light beams travel back into the SMF and interfere with each other owing to their different optical paths. The reflected light intensities are denoted as *I*_1_, *I*_2_, and *I*_3_, respectively. The cavity lengths (thickness of coating) of the first and second polymers are *L*_1_ and *L*_2_, respectively. The intensity of the reflected light measured using the principle of the multi-beam interference can be expressed as follows:

(1) $$\begin{array}{c} I=I_{1}+I_{2}+I_{3}-2\sqrt{I_{1}I_{2}}\;cos\left(\frac{4\pi }{\lambda }n_{p1}l_{2}+\varphi_{01}\right)+2\sqrt{I_{2}I_{3}}\;cos\left(\frac{4\pi }{\lambda }n_{p2}l_{1}+\varphi_{02}\right)\\ -2\sqrt{I_{1}I_{3}}\;cos\left(\frac{4\pi }{\lambda }\;\left(n_{p1}l_{2}+n_{p2}l_{1}\right)+\varphi_{03}\right) \end{array}$$

The wavenumber is $ῡ=\frac{2\pi }{\lambda }$, while $\varphi_{01}$*,* $\varphi_{02}$, and $\varphi_{03}$ are the respective initial phases. The phase factors for the 1st polymer cavity, 2nd polymer cavity, and both hybrid polymers (1st and 2nd polymers) can be expressed as follows for Equation (1):

(2) $$\varphi_{1}=ῡ\;\left(2n_{2}l_{2}\right)+\varphi_{01}$$

(3) $$\varphi_{2}=ῡ\;\left(2n_{1}l_{1}\right)+\varphi_{02}$$

(4) $$\varphi_{3}=ῡ\;\left(2n_{2}l_{2}+2n_{1}l_{1}\right)+\varphi_{03}$$

The discrete Fourier transform of Equation (1) uses the following formula:

(5) $$F\left(\xi_{i}\right)=\sum A_{n}(\xi_{i})\delta \left(\xi_{i}\right)$$

The optical paths of the three reflection surfaces are $\xi_{1}=2n_{2}l_{2}$, $\xi_{2}=2n_{1}l_{1}$, and $\xi_{3}=\xi_{1}+\xi_{2}$. The thickness of each polymer layer is responsible for the abscissa of the peak amplitudes. For comparison, different strategies were used for coating, and different polymer combinations were considered. Here, the 1st coated polymer was PMMA, which was common for all the considered combinations, whereas the 2nd coated polymer was either PC, PS, or polyacrylic acid (PAA).

### 2.2. Sensor Fabrication

**Scheme 2:**
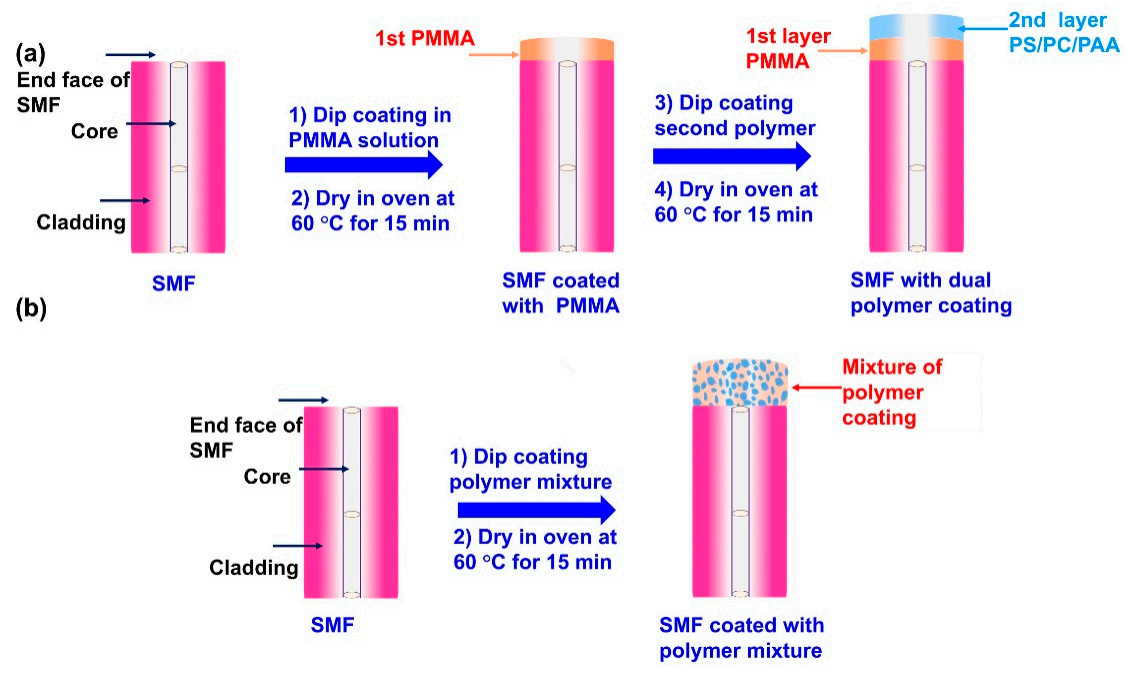
Schematic of (**a**) the fabrication of the stepwise DFPI-based sensors and (**b**) polymer-mixture-coated DFPI-based sensors.

The DFPI-based temperature sensor is shown in Scheme 1, whereas the DFPI-based sensor’s fabrication process is presented in Scheme 2. The used fiber was a standard SMF-28 (New York, NY, USA) with the cladding diameter of 125 µm and the core of 8.2 µm. The edge of the SMF tip was not flat, because the SMF cladding was covered with a very thin coating of zirconium oxide. It was established using the phase angle, which reduces the return loss and enables a uniform thin coating of the polymer. We considered three thermosensitive polymers, namely poly(methyl methacrylate) (PMMA) (Mw ~ 350,000, Sigma-Aldrich, Inc., St. Louis, MI, USA), polycarbonate (PC) (Sigma-Aldrich, Inc., St. Louis, MI, USA), and polystyrene (PS) (Mw ~ 350,000, Sigma-Aldrich, Inc., St. Louis, MI, USA). We also considered one non-thermosensitive polymer, namely PAA (Mw ~ 450,000, Sigma-Aldrich, Inc., St. Louis, MI, USA). These polymers were chosen owing to their good properties, including their transparent and colorless solution, high viscosity at low concentrations, and good adhesion to the SMF, as well as their auspicious optical properties, as listed in Table 1. When preparing high-quality sensors, the above superior characteristics of the underlying polymer are very important [1,23,24,33].

The DFPI-based sensors in this study were fabricated using two different dip-coating methods, utilizing SMF-28, as shown in Scheme 2. In this fabrication process, a 10 wt% solution of PMMA, PC, or PS was prepared in the calculated amount of chloroform (≥99.5%, Sigma-Aldrich, Inc., St. Louis, MI, USA) separately by simple dissolution through stirring. The prepared solutions were named 10 wt% PMMA, 10 wt% PC, and 10 wt% PS. The PAA solution (10 wt%) was prepared in ethanol (Duksan reagent, 99.9% pure, South Korea, Seoul) by stirring, to permit the total dissolution of the solid polymer and the creation of a viscous and homogeneous solution, which was named 10 wt% PAA. On the other hand, the polymer mixtures of the PMMA_PS, PMMA_PC, and PMMA_PAA were prepared as follows. First, 10 wt% solutions of all polymers were prepared separately, using the above method. Second, the two polymer solutions with a 1/1 (*v*/*v*) ratio were mixed by simple addition followed by stirring, and they generated mixed polymer solutions, which are referred to as PMMA_PS mixture, PMMA_PC mixture, and PMMA_PAA mixture. The first method for fabricating the DFPI-based sensors was a simple stepwise dip-coating method, as shown in Scheme 2a. In this method, the SMF tip was cleaned using isopropanol and dried at 27 °C. In the first step, the tip of the bare SMF was dipped into the PMMA solution for 5 min, to achieve good adherence of the polymer to the fiber, as well as to ensure air-bubble-free smooth coating. The dipped fiber was then placed in an oven, at 60 °C for 15 min, for drying. After achieving a thin, uniform, smooth, flat, and air-bubble-free PMMA coating, this sensor was used for the next step. In the second step, the PMMA-coated SMF tip was dipped into the PC solution for 5 min after being kept in the oven for 15 min, to dry the second layer of the polymer, and the obtained sensor was referred to as the PMMA_ PC-coated DFPI-based sensor. By following the same method, PMMA_PS and PMMA_PAA DFPI sensors were fabricated. The polymer mixture solution was coated at the tip of the SMF by simple dip coating, as shown in Scheme 2b. In the second fabrication method, the cleaned tip of the SMF was dipped in a mixture of PMMA_PC for 5 min, followed by drying in an oven at 60 °C for 15 min. The same process was repeated one more time to allow a desirable coating with the mixture on the SMF tip. The as-obtained sensor was referred to as the PMMA-PC-mixture-coated DFPI-based sensor. Following the same process, PMMA_PS-mixture and PMMA_PAA-mixture sensors were also prepared.

### 2.3. Measurement Setup 

**Figure 1:**
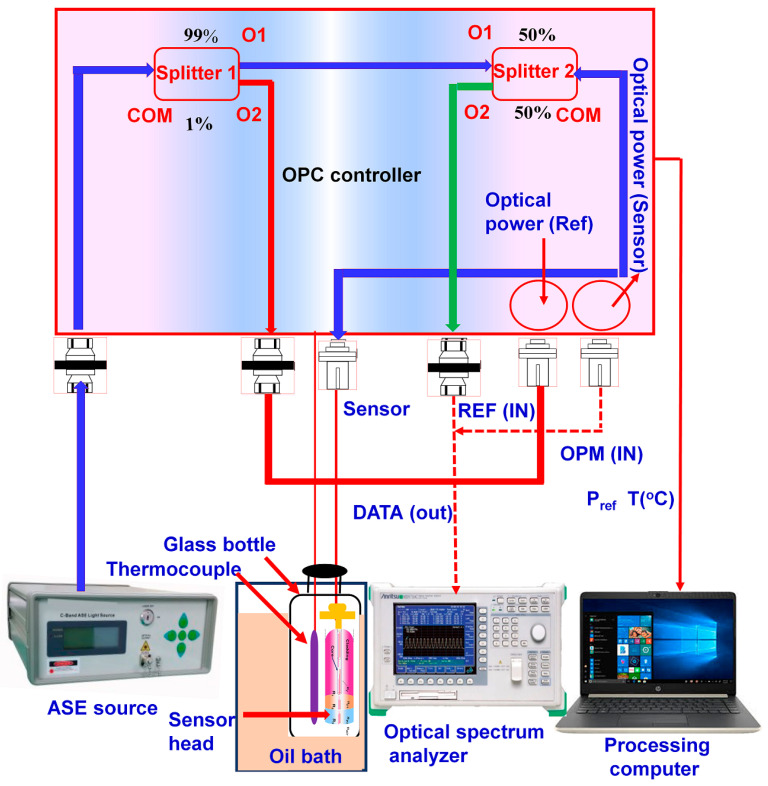
Configuration diagram of an experimental device for measuring the sensitivity of the DFPI-based temperature sensors.

The experimental setup for characterizing the fabricated Fresnel reflection-based temperature sensors is shown in Figure 1. The system was utilized for determining wavelength shifts with respect to the temperature change of the fabricated DFPI-based temperature sensors. The measurement system consisted of a C-band amplified spontaneous emission (ASE-BT-C-16-AF) broadband light source with the central wavelength of 1550 nm, an optical power meter containing an optical power controller (OPC), an optical spectrum analyzer (OSA) (Model MA9710C, Anritsu, Kanagawa Prefecture, Japan), a glass vial, an oil bath, a processing computer, and a tested DFPI-based sensor. The ASE light source supplied light to the OPC, which had two couples created by an optical fiber splitter; it was first split into splitter 1, and then divided into two parts at a 1:99% ratio. Consequently, the 1% part of the light was directly supplied to the OPC as a reference. The other end of the reference fiber was connected to the OSA to supply the reference signal. The 99% part of the light passed through directional splitter 2 and further split into two equal parts. One half of the light beam was transmitted to the DFPI-based temperature sensor, while the other half propagated directly to the OSA. The precise wavelength reflected from the sensor head reached splitter 2 and was detected by the OSA. The DFPI-based sensor was positioned with a thermocouple in the glass vial in the oil bath.

### 2.4. Temperature Response Test

The sensitivities of the fabricated DFPI-based sensors were evaluated in the wavelength shift experiment. To control the temperature, the sensor head and the thermocouple head (Giltron GT 307/08, New Taipei, Taiwan), with the resolution of ±0.1 °C, were introduced into the glass vial that was in the oil bath. Light injected from the ASE light source passed through the OPC, reaching the dual-polymer-coated sensor. The reflected light intensity was recorded by the OSA, and the spectra were computed and saved. The temperature of the oil bath was varied in 5 °C steps (except for the PMMA_PC DFPI sensor) in the ~24.4–80 °C range. Next, the temperature change was recorded by the thermocouple, and the corresponding spectral response was recorded by the OSA. Afterwards, the system was allowed to cool naturally from 80 to 24.4 °C. In addition, the spectral response change during the cooling was recorded. The same experiment was performed for all fabricated sensors in triplicate, and the average sensitivity was reported.

## 3. Results and Discussion

A high-quality DFPI-based sensor can be obtained if the coating material conforms to the Fresnel reflection criterion, according to which the RI of the coating material should be higher than that of the fiber core (silica, 1.456) and air (1.0) [23,33]. The RI differences between the coating material and SMF/air are responsible for the desirable fringe visibility of the reflected spectra; that is, the higher the difference, the higher the fringe visibility [34]. Specifically, we chose PMMA, PC, and PS owing to their thermosensitivity and relatively high refractive indices, which makes them useful for optic and fiber communication applications [23,33]. As per Equation (1), the sensing principle is typically based on the RI and thermal expansion properties of thermosensitive materials. The coating polymer acts as a microcavity that produces low-finesse interference through reflection from the surface (Scheme 1). The polymer coated on the tip of the SMF expands or constricts as a function of temperature, and the RI changes, altering the optical path and the phase difference between the successively reflected light beams [34,36]. The proposed DFPI-based sensor utilizes a simple three-beam interferometric mechanism. In the DFPI-based sensor, the optical path change and the resultant change in RI are higher in response to temperature, compared with those of a single polymer-coated sensor. This change creates a large phase difference for a small temperature change. Therefore, we fabricated our DFPI-based temperature sensors using stepwise dip coating and polymer mixture solution coating. Stepwise dip coating is a simple method for sensor fabrication. In addition to its simplicity, the coated polymers retain their physical and chemical properties, yielding promising sensors. On the other hand, polymer mixture coatings are not only difficult to achieve, but the physical and chemical properties of the constituent polymers may change after mixing, potentially compromising the quality of resultant sensors. PMMA, PC, and PS are renowned thermosensitive polymers with good TOCs and TECs (Table 1). However, PAA is a non-thermosensitive polymer; the optical path and RI cannot be temperature-modulated. All of the chosen polymers in the present study adhered well to the SMF, which is important for good DFPI-based sensors. As mentioned above, we fabricated seven sensors, using different polymer combinations.

**Figure 2:**
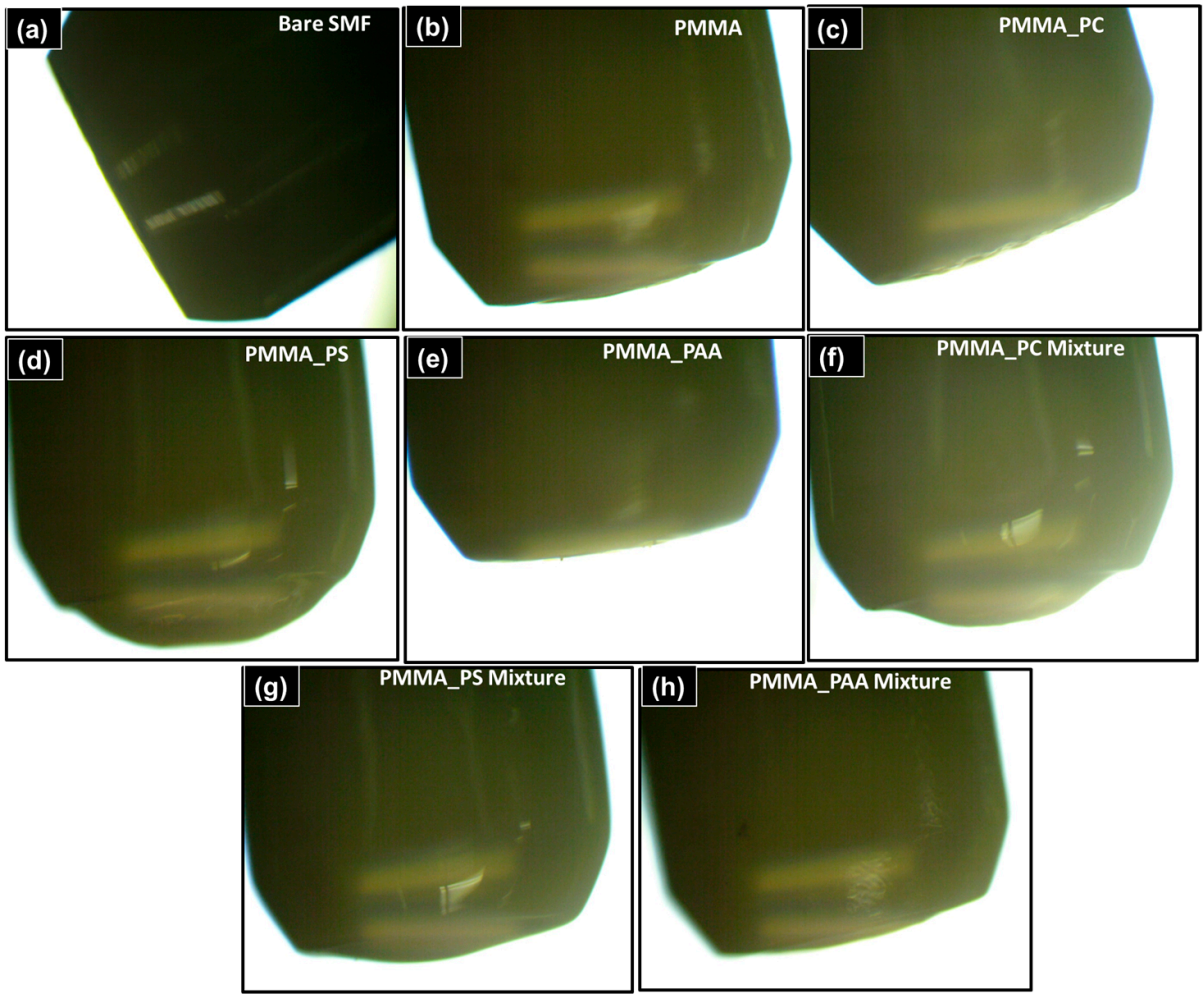
Optical microscopy images of (**a**) a bare SMF, and sensors coated with (**b**) PMMA, (**c**) PMMA_PC, (**d**) PMMA_PS, (**e**) PMMA_PAA, (**f**) PMMA_PC mixture, (**g**) PMMA_PS mixture, and (**h**) PMMA_PAA mixture.

**Figure 3:**
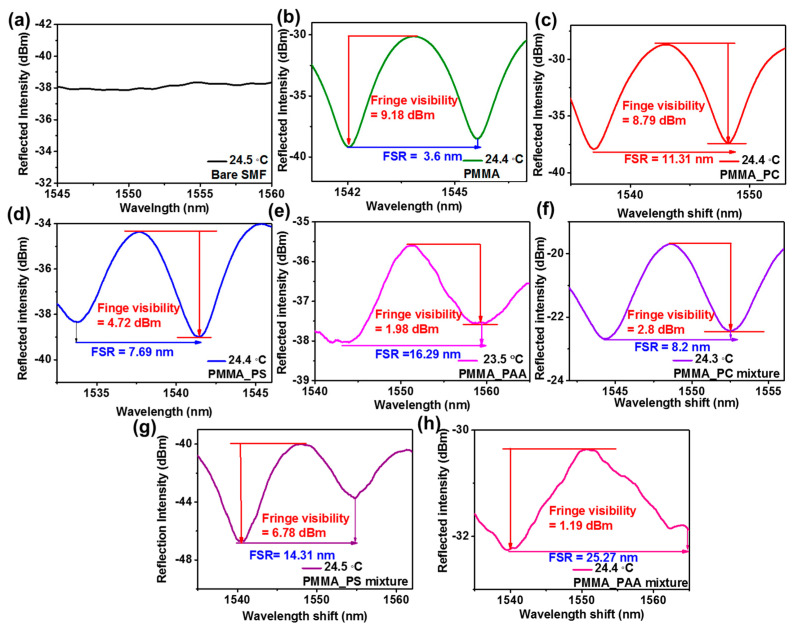
Reflection spectra of (**a**) a bare SMF, and of proposed sensors with (**b**) PMMA, (**c**) PMMA_PC, (**d**) PMMA_PS, (**e**) PMMA_PAA, (**f**) PMMA_PC mixture, (**g**) PMMA_PS mixture, and (**h**) PMMA_PAA mixture.

The morphology of the DFPI-based sensors was confirmed by microscopic images (MIC S16C, Microscopes INC, Winona Ave, St. Louis, MI, USA), which are shown in Figure 2. Figure 2a shows a microscopic image of the reference fiber (without polymer coating); the end phase is very clean, flat, and uniform. Figure 2b shows the side view of the PMMA-coated sensor; the formed microcavity is very thin, air-bubble-free, and uniform. Figure 2c–h show microscopic images of the PMMA_PC, PMMA_PS, PMMA_PAA, PMMA_PC-mixture, PMMA_PS-mixture, and PMMA_PAA-mixture sensors, respectively. The images prove that both the simple stepwise dip coating and polymer mixture solution coating methods yield air-bubble-free, thin, and uniform polymer-coated sensors. The adhesive properties of the coated polymers were also effective. As a result, the fabricated sensors were ready for temperature measurement tests. Sensors with non-uniform, rough coatings with air bubbles have compromised reflection spectra [35]. The reflected interference pattern of the reference SMF corresponds to a straight line (Figure 3a), which indicates that most of the light was communicated through the end phase of the SMF. Figure 3b–h show the reflection spectra of the PMMA, PMMA_PC, PMMA_PS, PMMA_PAA, PMMA_PC-mixture, PMMA_PS-mixture, and PMMA_PAA-mixture sensors, at room temperature (~24.4 °C), with free spectral ranges (FSRs) of 3.6, 11.31, 7.69, 16.29, 8.2, 14.31, and 25.27 nm, respectively. Large microcavity sensors have denser interference patterns, whereas thinner microcavity sensors have rarer interference patterns. The spectra depend on various factors, such as the fabrication method, the polymers, properties, RI, geometry of coating, and length of the microcavity [33,34]. Among them, the most important one is human-incurred unpredictability, but it is also very important to consider the geometry of the coating, the polymers, properties, RI, and length of the microcavity, all of which affect the visibility of the interference pattern. For checking the reproducibility of the sensors, we prepared them with the same method, using which they demonstrated the same reflected spectra for the PMMA, PMMA_PC, and PMMA_PS sensors as shown in Figure S1. The result illustrates that the FSR and fringe visibility are much closer to those of the previously fabricated sensors, and the spectra is very smooth as well. Therefore, we conclude that the coating is uniform and air-bubble-free and produces approximately the same thickness, and that the applied coating method is reproducible. The PMMA_PAA sensor exhibited very low fringe visibility (1.98 dBm) and rough spectra, which could be attributed to the non-thermosensitive property and optical properties of PAA, owing to the weak Fresnel reflection at the PAA/air interface [34]. Likewise, the polymer mixture sensors exhibited poor fringe visibility and rough spectra, owing to the change in the optical properties of the original polymer after mixing (Figure 3f–h and Table 2). For instance, the PMMA_PC (8.79 dBm) and PMMA_PS (4.72 dBm) sensors exhibited high fringe visibility and excellent spectral performance, suggesting good quality on account of the intensity of the three interference beams. The Fresnel reflection at the PC/air and PS/air interfaces was stronger than that at the other two interfaces, which was further supported by Equation (1). The results illustrate that stepwise dip coating is promising in terms of achieving high-quality sensors.

**Figure 4:**
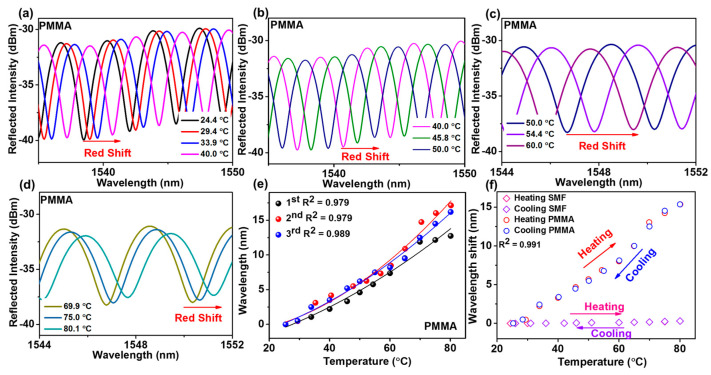
(**a**–**d**) Reflection spectra of the PMMA-coated sensors at different temperatures (exhibiting a red shift). (**e**) Wavelength shift for three measurements. (**f**) Average wavelength shift of the PMMA sensor vs. temperature.

**Figure 5:**
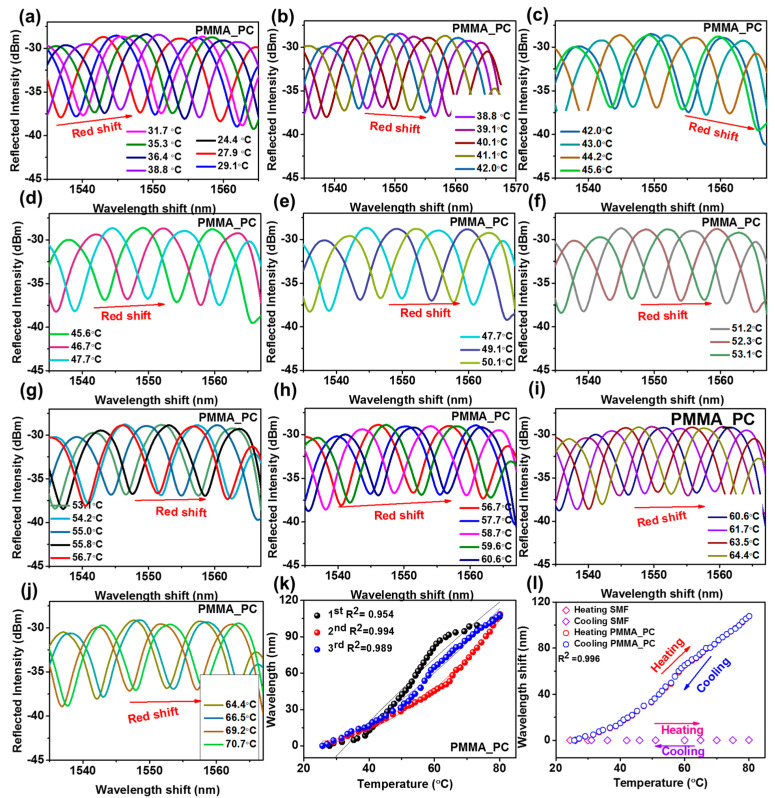
(**a**–**j**) Reflection spectra of the PMMA_PC-coated sensor for different temperatures (demonstrating the red shift). (**k**) Wavelength shifts for three measurements and (**l**) Average wavelength shift of the PMMA_PC sensors.

The proposed sensors were used for measuring the temperature of their environments in order to evaluate their performance. The sensor end was placed inside a glass vial with a thermocouple, and the vial was kept in an oil bath. The temperature was increased from room temperature (24.4 °C) to 80 °C in steps of ~5 °C, and the reflection spectra obtained as a result of the temperature change were recorded for all sensors; the results are summarized in Figure 4, Figure 5 and Figures S2–S6 in the Supporting Information. The reflection spectra of all the sensors exhibited a red shift (a shift toward longer wavelengths) as the temperature increased (Figure 4, Figure 5, and Figures S3 and S4), whereas a blue shift (a shift toward shorter wavelengths) was observed as the temperature decreased (Figures S2, S3 and S6). The wavelength shift originated from the change in the RI (owing to the TOC change) and the change in the diaphragm thickness (owing to the TEC change) of the sensors [2,36].

The average sensitivity of the PMMA-coated sensor, over three measurements, was 279.5 pm °C^−1^. The PMMA_PC-and PMMA_PS-coated sensors exhibited considerably high average temperature sensitivities of 2142.5 and 787.5 pm °C^−1^ for temperatures in the 24.4–80 °C range. Note that single polymer-coated sensors cannot yield large optical path changes in response to small temperature changes, owing to their limited elastic moduli. In contrast, dual-polymer-coated sensors can exhibit larger optical path changes at their interfaces. For instance, the high values of TOC and TEC for the PMMA and PC had some differences, which might have generated synergistic effects to enhance the change in the optical path resulting from the optical properties of the two constituent polymers. As a result, they showed significant responses to small temperature variations, thus exhibiting very high sensitivity. For comparison, the PMMA_PAA-coated sensor was considered for two scenarios, one of which featured a thermosensitive polymer while the other one featured a non-thermosensitive polymer. The average sensitivity was ~198.4 pm °C^−1^. The PMMA_PAA-coated sensor exhibited lower sensitivity than the single PMMA-coated sensor, which could be attributed to the non-thermosensitive nature of the PAA polymer, which restricted the change in the optical path in response to a temperature change. To determine the response of the polymer-mixture-coated sensors, PMMA_PC-mixture-, PMMA_PS-mixture-, and PMMA_PAA-mixture-coated sensors were considered under the same environmental conditions. The temperature sensitivities of these three sensors were 339.5, 233.0, and 164.6 pm °C^−1^, for the PMMA_PC-mixture, the PMMA_PS-mixture, and the PMMA_PAA-mixture sensors, respectively. The results suggest that mixture-coated sensors have lower sensitivity than stepwise dual-layer-coated sensors, which could be due to the loss of optical properties caused by the formation of the polymer mixture. Thus, the polymer coating method importantly affects the quality and sensitivity of sensors.

To examine the sensors’ stability and temperature response, measurements were performed for each sensor in triplicate, and the resulting relationships between temperature and wavelength shift are plotted in Figure 4e, Figure 5k, Figures S3c,f,j and S4b,e, for the PMMA, PMMA_PC, PMMA_PS, PMMA_PAA, PMMA_PC-mixture, PMMA_PS-mixture, and PMMA_PAA-mixture sensors, respectively. The results suggest that the sensors’ responses were appropriately fitted by second-degree polynomials, which suggests the feasibility of the experiments and the proportional relationship between the temperature and wavelength shift, with good stability. Spectral shifts were observed, owing to the thermal expansion and thermo-optic properties of the coated polymers, for temperatures in the ~24.4–80 °C range. Average wavelength shifts (over three measurements) for temperatures in the ~24.4–80 °C range were calculated and are presented in Figure 4f and Figure 5l, for the PMMA, PMMA_PC, PMMA_PS, PMMA_PAA, PMMA_PC-mixture, PMMA_PS-mixture, and PMMA_PAA-mixture sensors, respectively. All of the considered sensors were well fitted by second-degree polynomials, with the goodness of fit coefficients of R2 = 0.991, 0.996, 0.999, 0.983, 0.991, 0.991, and 0.981, for the PMMA, PMMA_PC, PMMA_PS, PMMA_PAA, PMMA_PC-mixture, PMMA_PS-mixture, and PMMA_PAA-mixture sensors, respectively. The stepwise dip-coated sensors (PMMA_PC, PMMA_PS) exhibited better feasibility than the mixture and single-coated polymer sensors. However, the stepwise-coated PMMA_PAA sensor exhibited a lower goodness of fit (0.983), which could be due to the non-thermosensitive nature of PAA. In addition, the standard deviations of the sensors’ sensitivities were calculated and are presented in Table 2. The measured standard deviations were 0.041, 0.047, 0.012, 0.057, 0.049, 0.018, and 0.0073, for the PMMA, PMMA_PC, PMMA_PS, PMMA_PAA, PMMA_PC-mixture, PMMA_PS-mixture, and PMMA_PAA-mixture sensors, respectively. These results confirm that the designed DFPI-based sensors have excellent stability and reproducibility.

**Figure 6:**
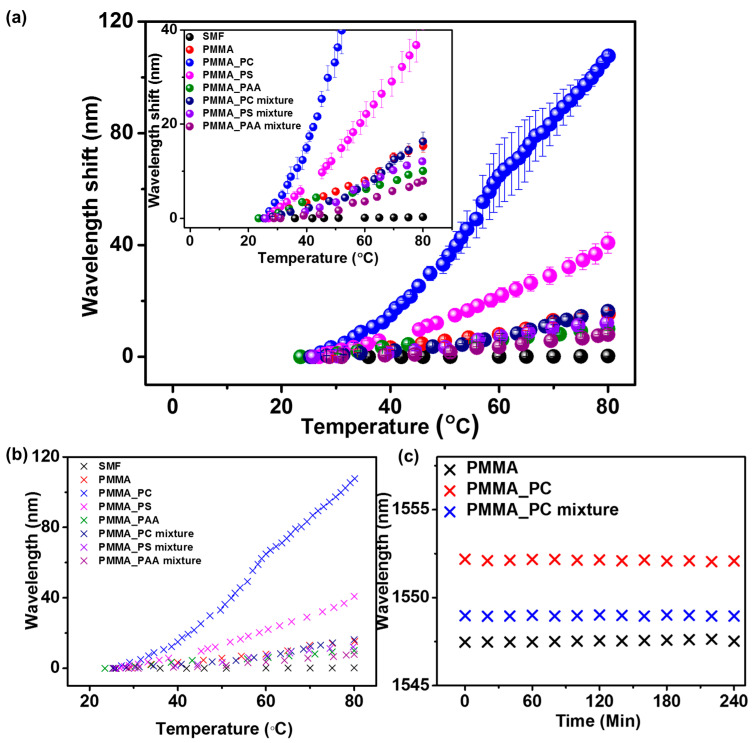
(**a**) Average wavelength shifts for all sensors. Inset is the zoomed image. (**b**) Sensitivities of all sensors compared with that of the bare SMF. (**c**) Wavelength fluctuations for the PMMA, PMMA_PC, and PMMA_PC mixture sensors, assessed over 4-h-long recording time.

The temperature response of the bare SMF was analyzed and compared with those of the other sensors, in terms of their standard error bars, and the results are shown in Figure 6a. The spectral shift for the bare SMF as a function of temperature was not considerable, whereas the spectral shifts for the proposed DFPI-based sensors were prominent. Specifically, the stepwise dip-coated PMMA_PC (2142.5 pm °C^−1^) and PMMA_PS (787.5 pm °C^−1^) sensors exhibited very high sensitivity compared with the other sensors, as discussed above. The PMMA_PC-coated sensor exhibited better sensitivity than the PMMA_PS-coated sensor, which could be due to the coating thickness in addition to the synergistic effect, as previously discussed. The thickness of the polymer coating is critical for achieving high-sensitivity sensors, as reported previously. Thin coatings typically yield high-sensitivity sensors [20,33,34,36]. When comparing the results shown in Figure 2c,d), it is evident that the PMMA_PC sensor has a very thin coating compared with the PMMA_PS sensor (the viscosities of PC and PS are different). Additional evidence for this difference is in the FSR values of PMMA_PC (FSR = 11.3 nm) and PMMA_PS (FSR = 7.7 nm). In a thin-coated sensor, the volume expansion of the polymer may be less restricted; consequently, the optical path can be significantly altered, yielding a higher spectral shift than that exhibited by thick-coated sensors. Meanwhile, temperature-incurred changes in the sensors’ spectra illustrate their better sensitivity at high temperatures. Figure 6a,b show that the wavelength shift in the proposed sensors is strongly associated with temperature. The dual-polymer-coated sensor exhibited excellent sensitivity at high temperatures (~70–80 °C). The sensitivities were, respectively, 355.86, 2279.35, 1055.74, 207.43, 529.43, 235.85, and 228.67, for the PMMA, PMMA_PC, PMMA_PS, PMMA_PAA, PMMA_PC-mixture, PMMA_PS-mixture, and PMMA_PAA-mixture sensors. The corresponding wavelength shift for the PMMA_PC sensor was the highest among them (refer to Figure S7 and Table 2), which was almost 227-fold that of the ordinary fiber Bragg grating-based sensor, and nearly 27.3-fold that of the long-period grating-based sensor [1,34]. Therefore, compared with other reported results (Table S1), stepwise dip-coated dual-polymer sensors consisting of two thermosensitive polymers exhibit superior sensing performance, better reproducibility, are simple to fabricate, and do not require sophisticated instruments/techniques. Finally, the sensors were evaluated for their stability, and the reflection spectra of the sensors in the 1550 nm band were recorded for the PMMA, PMMA_PC, and PMMA_PC mixture sensors, as a function of time, at a constant temperature (~24.4 °C) for 4 h. The results are shown in Figure 6c and Figure S8. The standard deviations of the measured temperature (Table S2) during the 4-h-long recording were 0.067, 0.070, and 0.063, for the PMMA, PMMA_PC, and PMMA_PC mixture sensors, respectively. The standard deviations of the wavelengths were 0.056, 0.040, and 0.023, for the PMMA, PMMA_PC, and PMMA_PC mixture sensors, respectively, suggesting high stability.

## 4. Conclusions

Dual-polymer-coated Fabry–Perot interferometer (DFPI)-based temperature sensors were proposed by utilizing two different coating methods: the stepwise dip-coating method and the polymer mixture-based coating method. Among them, the stepwise dip-coating method enabled a simple and reproducible preparation, providing high sensitivity, feasibility, and good stability. The PMMA_PC sensor exhibited the maximal temperature sensitivity of 2142.5 pm °C^−1^, based on the spectral shift measurements, which was ~7.7-fold that of the PMMA-coated sensor. The excellent performance was attributed to the fact that the two polymers were thermosensitive with high different TOC and TEC values, resulting in the significant response to small temperature variations, and the coating was very thin, uniform, and air-bubble-free. The dual polymer facilitated a significant optical path change compared with that enabled by single polymer-coated sensors. As a result, a large spectral shift was observed for a small change in temperature. The sensors that combined thermosensitive (PMMA) and non-thermosensitive (PAA) materials did not exhibit good performance because PAA is a non-thermosensitive polymer, which restricts the volume expansion of PMMA. The polymer-mixture-coated DFPI-based sensors also exhibited low sensitivity, which could be attributed to a change in their original optical properties. All stepwise dip-coated sensors were fitted well by second-degree polynomials for temperatures in the 24.4–80 °C range, and exhibited good reproducibility and stability. In summary, the proposed DFPI-based sensors, specifically the sensors with stepwise double thermosensitive polymer coating, exhibited high sensitivity, simplicity of fabrication, and low cost (around 10 USD). Thus, these sensors are promising for biomedical, biochemical, and physical temperature-sensing applications.

## Figures and Tables

**Table 1:** Properties of the selected polymers and used solvents.

Polymer	Refractive Index	TOC (°C^−1^)	TEC (°C^−1^)	T_g_ (°C)	Used Solvent for 10 wt%	Ref.
PMMA	1.48	−1.3 × 10^−4^	2.2 × 10^−4^	80–105	Chloroform	[33,37]
PC	1.585	−0.9 × 10^−4^	1.7 × 10^−4^	145	Chloroform	[33,37]
PS	1.59	−1.2 × 10^−4^	2.2 × 10^−4^	100	Chloroform	[33,37]
PAA	1.527	-	-	75–126	Ethanol	

**Table 2:** Comparison of FRS, fringe visibility, average sensitivity, standard deviation of sensitivity, regression coefficient, and wavelength shift with temperature steps, for different DFPI-based sensors.

Sensor	FSR (nm)	Fringe Visibility (dBm)	Average Temperature Sensitivity (pm °C^−1^) 24.4–80 °C	Standard Deviation of the Sensitivity (pm °C^−1^)	Average Temperature Sensitivity (pm °C^−1^) ~70–80 °C	Confidence Factor (R^2^)	Wavelength Shift (nm)	Wavelength Shift in Interval of Temperature (°C)
PMMA	3.6	9.18	279.5	0.0407	355.86	0.991	2.25	8.1
PMMA_PC	11.3	8.79	2142.5	0.0469	2279.35	0.996	5.31	1.1
PMMA_PS	7.7	4.7	787.5	0.0121	1055.74	0.999	4.4	6.6
PMMA_PAA	16.3	1.98	198.42	0.057	207.43	0.983	3.33	12.5
PMMA_PC mixture	8.2	2.8	339.5	0.049	529.43	0.991	0.99	7.1
PMMA_PS mixture	14.3	6.8	233.0	0.018	235.85	0.991	2.21	6.0
PMMA_PAA mixture	25.3	1.2	164.6	0.0073	228.67	0.981	5.79	11.4

## Data Availability

The data presented in this study are available on request from the corresponding author.

## Supplementary Materials

The following are available online at https://www.mdpi.com/article/10.3390/s21227632/s1, Figure S1: Reflected spectra for PMMA@R (a), PMMA_PC@R (b) and PMMA_PS@R (c) sensors for reproducibility. Figure S2: Blue shift reflection spectra of PMMA; Figure S3: Blue shift reflection spectra of PMMA-PC; Figure S4: Reflection spectra of PMMA-PS, PMMA-PAA, and PMMA-PC mixture; Figure S5: Reflection spectra of PMMA-PS mixture and PMMA-PAA mixture; Figure S6: Blue shift reflection spectra of PMMA-PS, PMMA-PAA, PMMA-PC mixture, PMMA-PS mixture, and PMMA-PAA mixture; Figure S7: Comparison of the wavelength shift of the FPI sensors; Figure S8: Wavelength of spectral dip response at a constant temperature; Table S1: Comparison of sensitivities and preparation methods/complication of various fiber optic temperature sensors; Table S2: Comparison of the wavelength fluctuation for PMMA, PMMA_PC and PMMA_PC mixture sensors.
