# Supplementary material for: Simply Fabricated Inexpensive Dual-Polymer-Coated Fabry-Perot Interferometer-Based Temperature Sensors with High Sensitivity
Source: Sensors (Basel). 2021 Nov 17;21(22):7632. doi: 10.3390/s21227632 (PMC8620928; doi:10.3390/s21227632)
Supplement: Supplementary file 1 [file sensors-21-07632-s001.zip › sensors-1456097-supplementary.pdf]

sensors

Supplementary

Simply Fabricated Inexpensive Dual-Polymer-Coated FABRY-Perot Interferometer-Based Temperature Sensors with High Sensitivity

Tejaswi Tanaji Salunkhe [1,†], Ho Kyung Lee [1,†], Hyung Wook Choi [2], Sang Joon Park [1] and Il Tae Kim [1,*]

[1] Department of Chemical and Biological Engineering, Gachon University, Seongnam-si, 13120 Gyeonggi-do Korea; tejaswisalunkhe235@gmail.com (T.T.S.); ghrud0722@gmail.com (H.K.L.); psj@gachon.ac.kr (S.J.P.)
[2] Department of Electrical Engineering, Gachon University, 13120 Seongnam-si, Gyeonggi-do, Korea; chw@gachon.ac.kr
* Correspondence: itkim@gachon.ac.kr; Tel.: +82-31-750-8835; Fax: +82-31-750-5363
† Those authors equally contributed to this work.

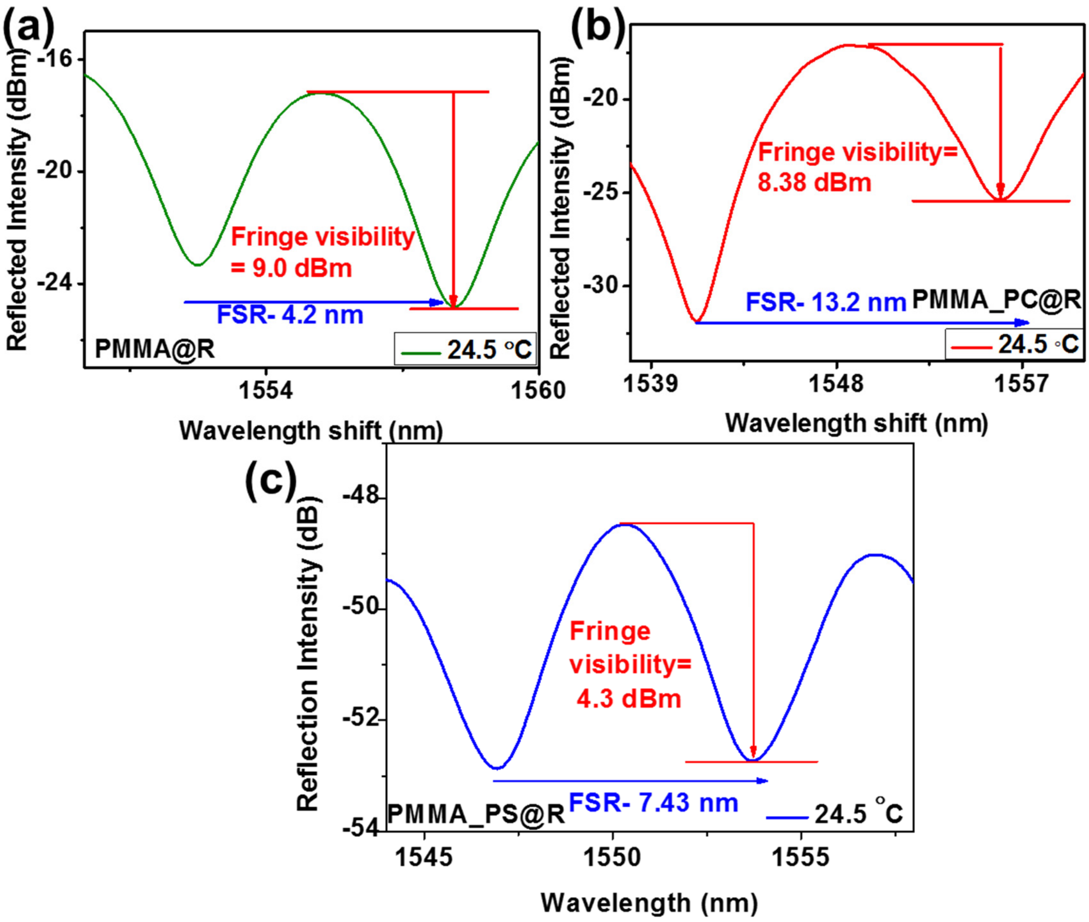

Figure S1. Reflected spectra for (**a**) PMMA@R, (**b**) PMMA_PC@R, and (**c**) PMMA_PS@R sensors for checking reproducibility.

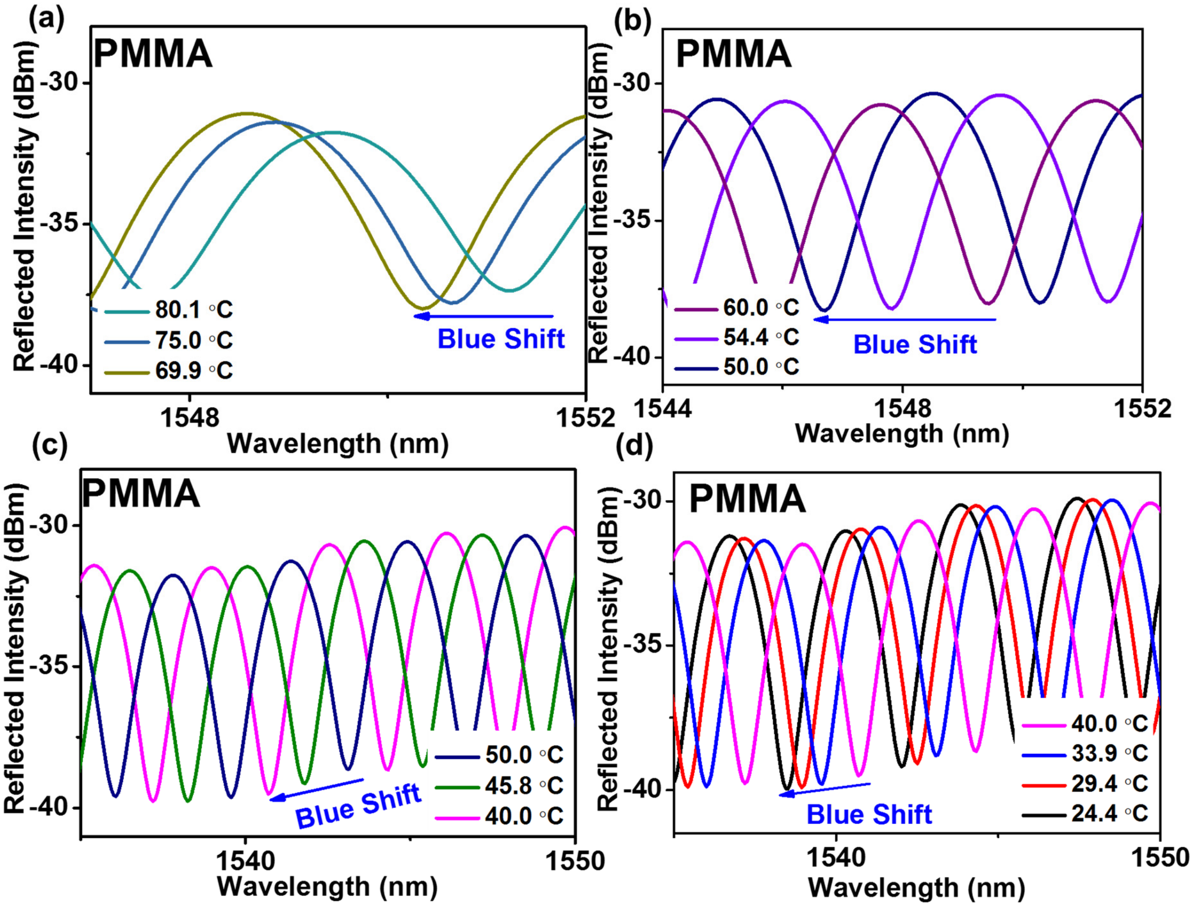

Figure S2. (**a**-**d**) Reflection spectra of the PMMA coated sensor in response to a temperature decrease (Blue shift).

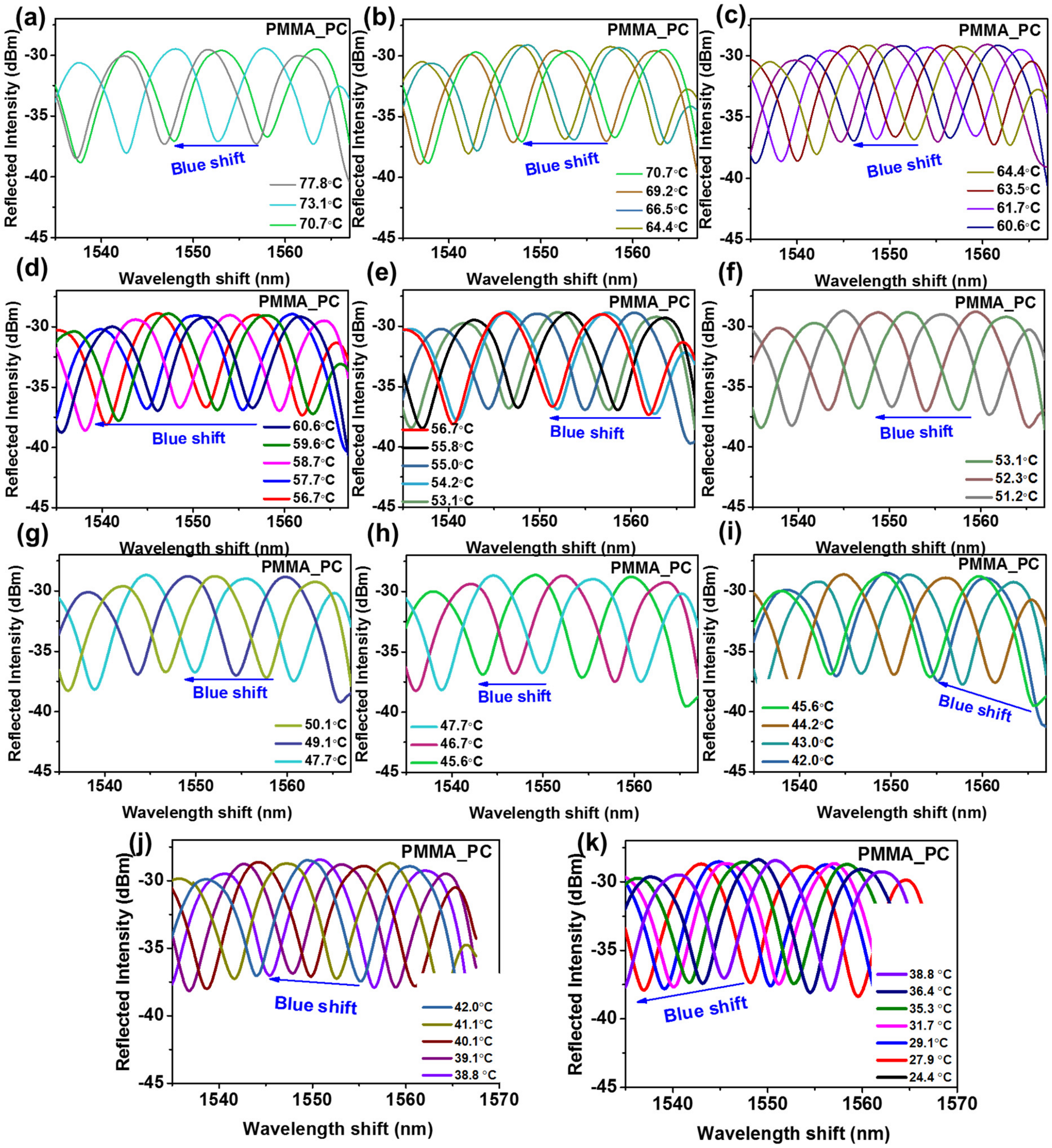

Figure S3. (**a**-**k**) Reflection spectra of the PMMA_PC coated sensor in response to a temperature decrease (Blue shift).

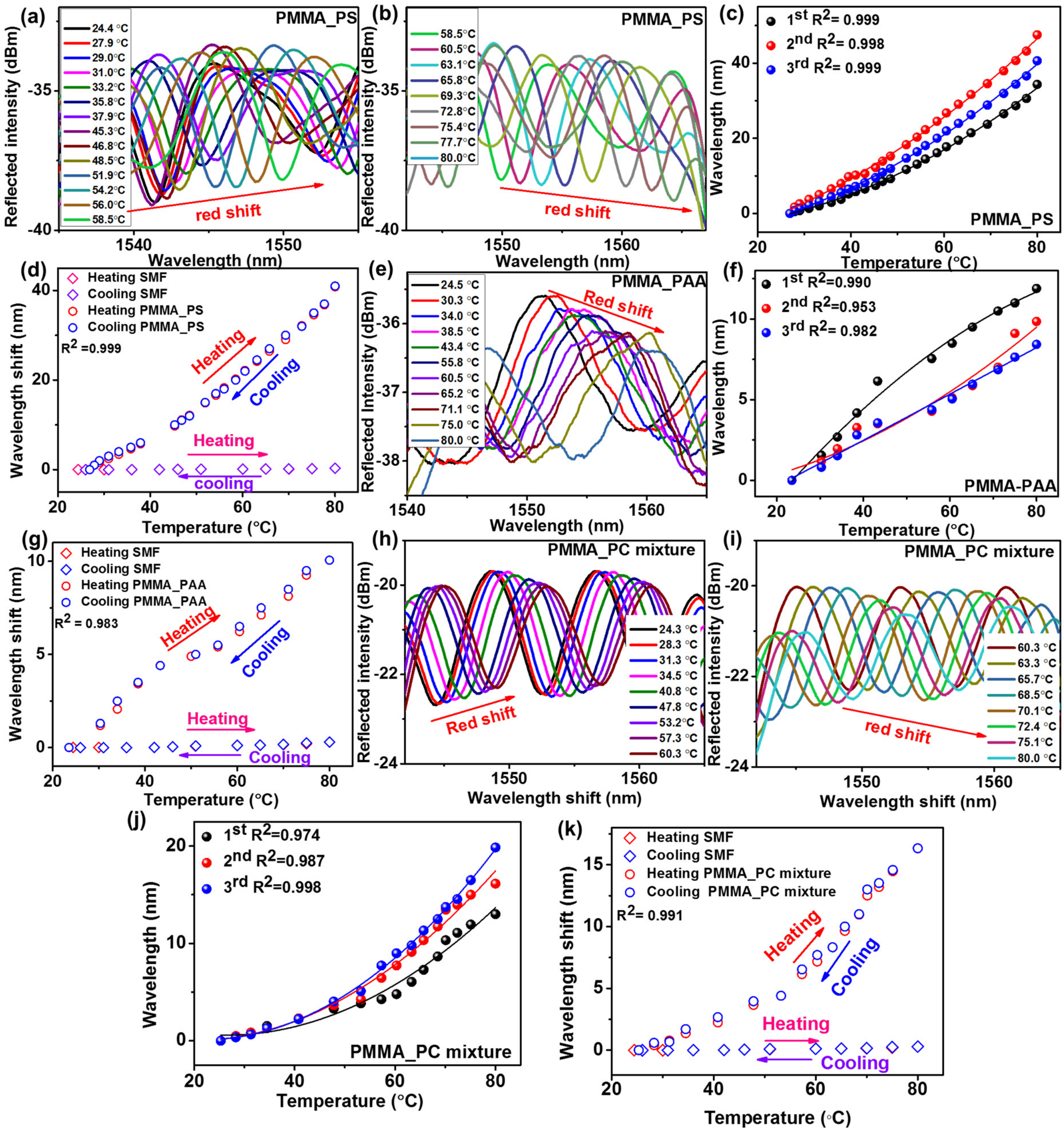

Figure S4. (**a**-**b**) Reflection spectra of the PMMA_PS coated sensor in response to a temperature increase (red shift). (**c**) wavelength shift for three measurements and (**d**) average wavelength shift of PMMA-PS sensor. (**e**) reflection spectra of the PMMA_PAA coated sensor in response to a temperature increase (red shift). (**f**) wavelength shift for three measurements and (**g**) average wavelength shift of PMMA-PAA sensor. (**h**-**i**) reflection spectra of the PMMA_PC mixture coated sensor in response to a temperature increase (red shift). (**j**) wavelength shift for three measurements and (**k**) average wavelength shift of PMMA-PC mixture sensor.

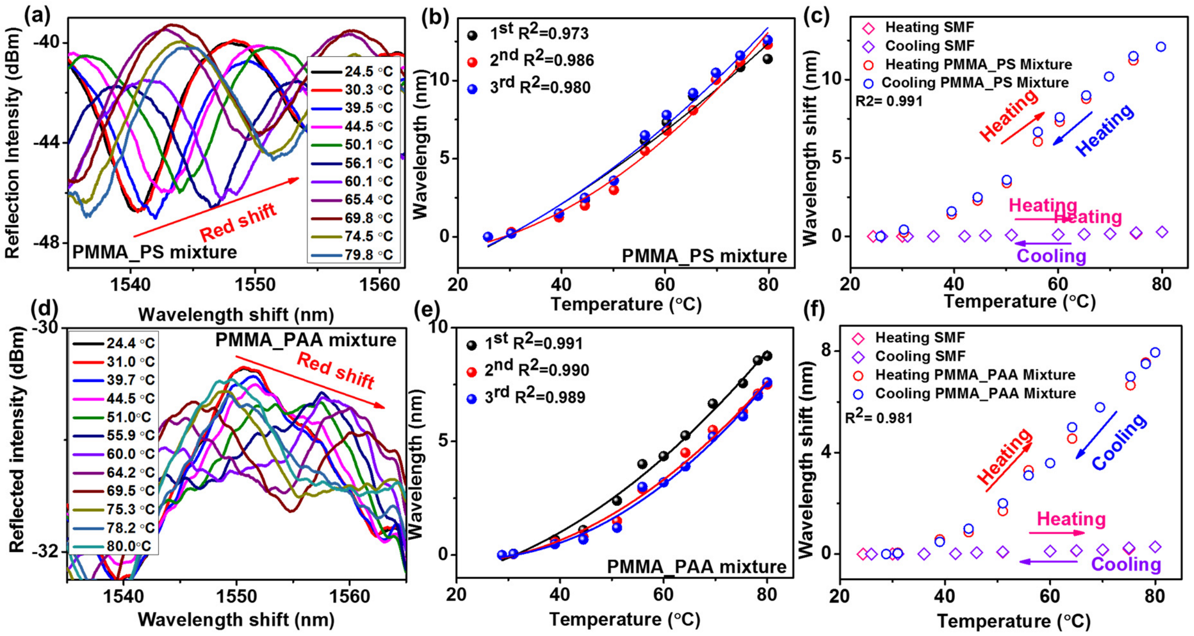

Figure S5. (**a**) Reflection spectra of the PMMA_PS mixture coated sensor in response to a temperature increase (red shift). (**b**) wavelength shift for three measurements and (**c**) average wavelength shift of PMMA-PS mixture sensor. (**d**) reflection spectra of the PMMA_PAA mixture coated sensor in response to a temperature increase (red shift). (**e**) wavelength shift for three measurements and (**f**) average wavelength shift of PMMA-PAA mixture sensor.

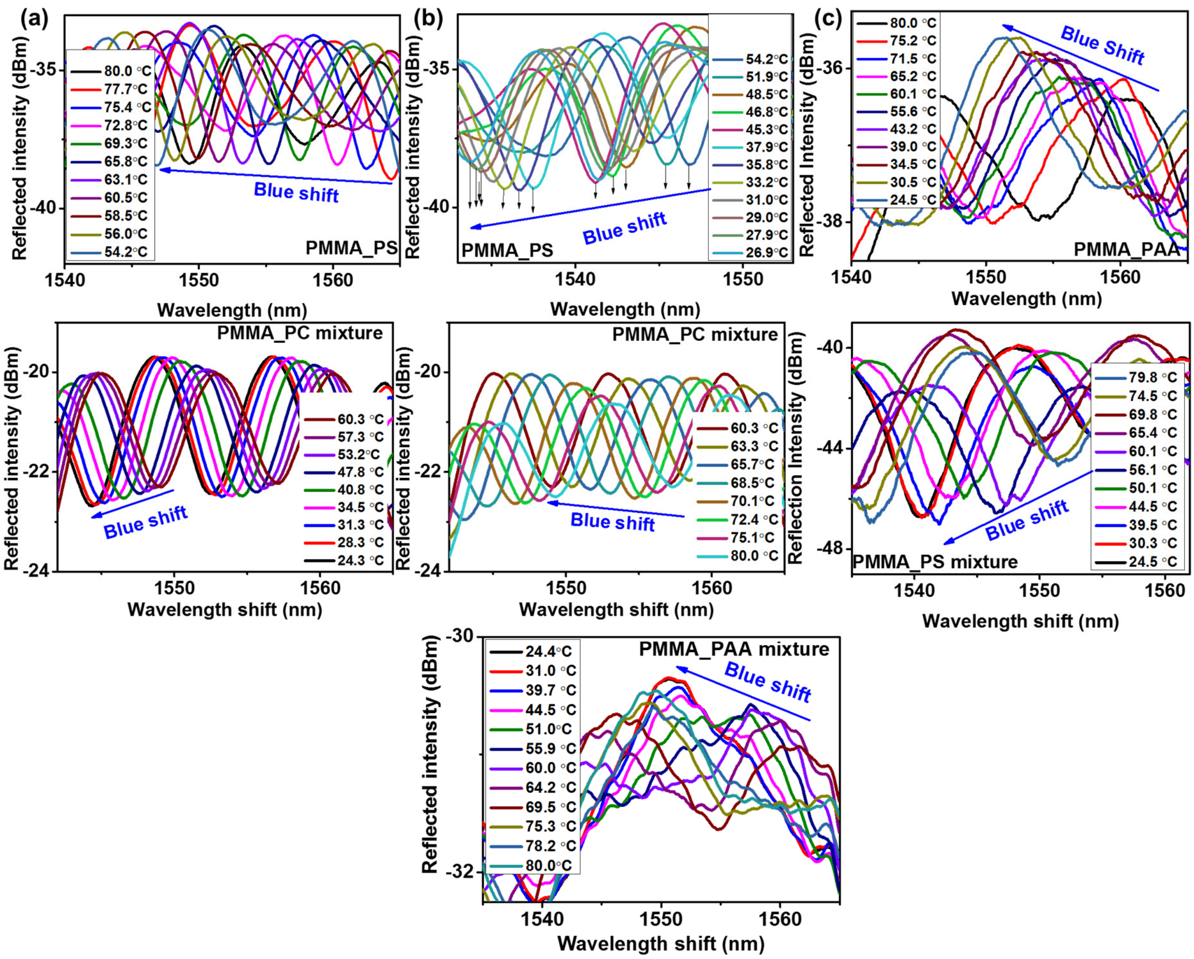

Figure S6. The reflection spectra of in response to a temperature decrease (blue shift) (**a**-**b**) PMMA_PS, (**c**) PMMA_PAA, (**d**-**e**) PMMA_PC mixture, (**f**) PMMA-PS mixture, and (**g**) PMMA_PAA mixture. .

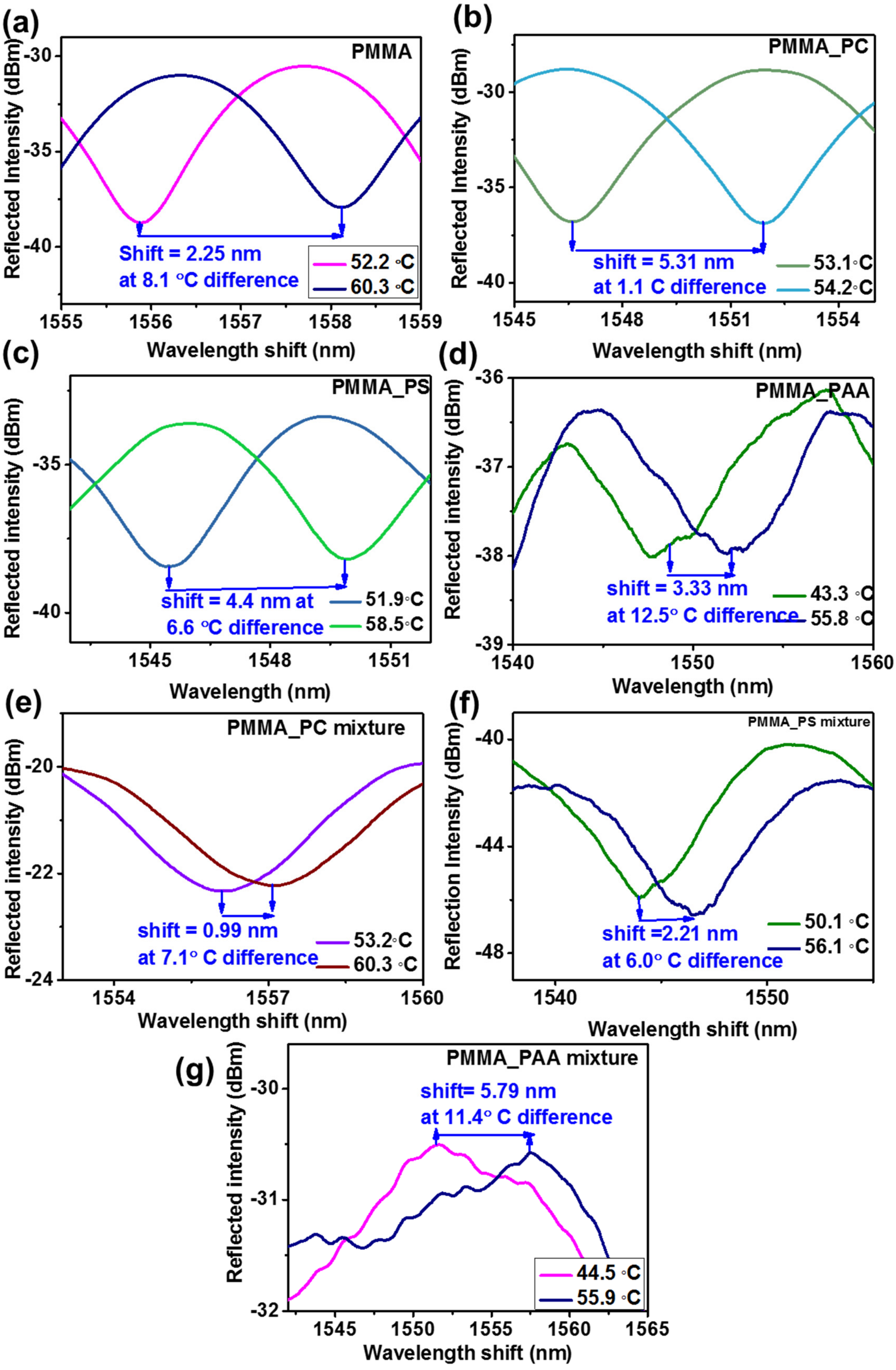

Figure S7. Comparison of the wavelength shift of FPI sensor at a particular temperature range.

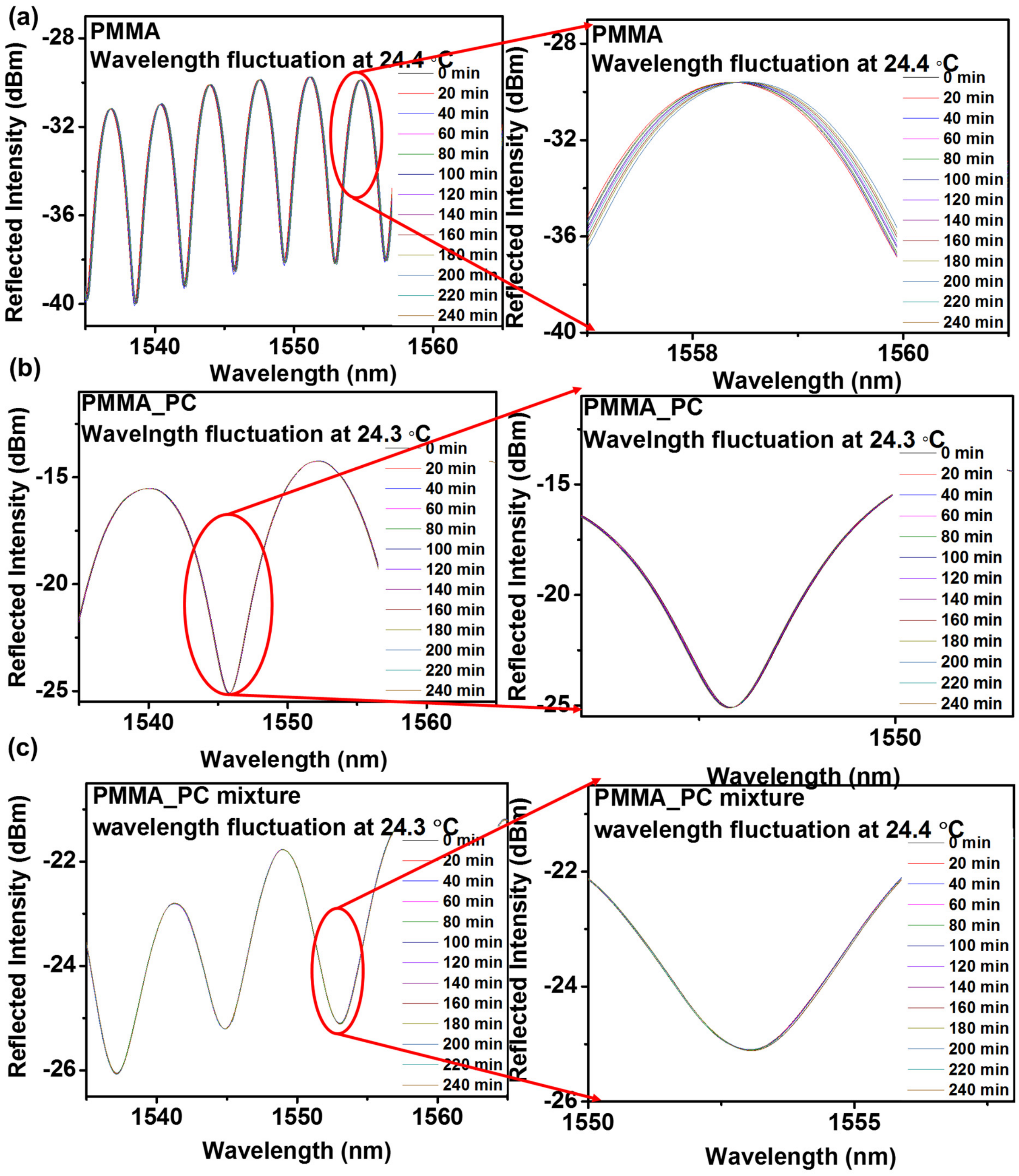

Figure S8. Wavelength of spectral dip response at a constant temperature and at various time intervals of (a) PMMA, (b) PMMA_PC and PMMA-PC mixture.

Table S1. Comparison of sensitivities and preparation methods/complication of various fiber optic temperature sensors.

Type of Fiber	Polymer	T(°C)	Sensitivity (pm °C^{-1})	Method	Ref
Fabry–Perot Interferometer	polyvinyl alcohol	25-100	~193.3	Low sensitivity, Stepper motor needed for coating	[1]
Fiber Bragg grating	Norland Optic Adhesive-61	10-50	19.5	Operation temperature range is small, UV curing method needed for coating the polymer	[2]
Fiber Fizeau interferometer	Norland Optic Adhesive-61	10-50	269.5	Operation temperature range is small, UV curing method needed for coating the polymer	[2]
Fabry–Perot Interferometer	Poly (vinyl chloride)	25-60	366.0	Plastic welder used for coating, low sensitivity	[3]
Single mode + Hollow core fiber	polydimethylsiloxane	51-70.5	2703.5	expensive method, poor reproducibility, requires expensive techniques, complex method	[4]
Microfiber mode interferometer	polydimethylsiloxane	20-48	3101.7	requires expensive techniques,	[5]
Fabry–Perot Interferometer	step-curing ultraviolet photoresist and polydimethylsiloxane	20-75	689.68	Low sensitivity, UV curing method needed for coating	[6]
Fabry–Perot Interferometer	Polycarbonate	20-140	245.4	Low sensitivity,	[7]
Fabry–Perot Interferometer	Polystyrene	25-100	439.89	Low sensitivity,	[8]
Fiber Bragg grating	gold-coated shallow-tapered chirped	30-80	9.893	CO_2 laser splicing system, Gold Layer Sputtering, optical backscatter reflectometer interrogator	[9]
Fiber Bragg grating +Single mode +Multimode Fiber	–	0-900	13.4	femtosecond laser inscription, fusion splicer	[10]
Ultra-long period fiber grating + graded index multimode fiber	doping of germanium	30-150	90.77	Splicing by arc discharge, fusion splicer	[11]
**Fabry–Perot Interferometer**	**PMMA_PC**	**24.4-80**	**2142.5**	**Simple , cheap, reproducible, high sensitivity**	**This work**
**Fabry–Perot Interferometer**	**PMMA_PS**	**24.4-80**	**785.5**	**Simple , cheap, reproducible, high sensitivity**	**This work**

Table S2. Comparison of the wavelength fluctuation for PMMA, PMMA_PC and PMMA_PC mixture sensors.

Sensor	Average Temperature for Wavelength Fluctuation (°C)	Standard Deviation for Temperature (°C)	Standard Deviation for Wavelength (nm)
PMMA	24.4	0.0674	0.0555
PMMA_PC	24.3	0.0699	0.0402
PMMA_PC mixture	24.3	0.0632	0.0239

References

1. Q. Rong, H. Sun, X. Qiao, J. Zhang, M. Hu, Z. Feng, A miniature fiber-optic temperature sensor based on a Fabry–Perot interferometer, Journal of Optics, 14 (2012) 045002.
2. C.-L. Lee, Y.-W. You, J.-H. Dai, J.-M. Hsu, J.-S. Horng, Hygroscopic polymer microcavity fiber Fizeau interferometer incorporating a fiber Bragg grating for simultaneously sensing humidity and temperature, Sensors and Actuators B: Chemical, 222 (2016) 339-346.
3. Z. Zhang, C. Liao, J. Tang, Z. Bai, K. Guo, M. Hou, J. He, Y. Wang, S. Liu, F. Zhang, Y. Wang, High-Sensitivity Gas-Pressure Sensor Based on Fiber-Tip PVC Diaphragm Fabry–Pérot Interferometer, Journal of Lightwave Technology, 35 (2017) 4067-4071.

4. M.-q. Chen, Y. Zhao, F. Xia, Y. Peng, R.-j. Tong, High sensitivity temperature sensor based on fiber air-microbubble Fabry-Perot interferometer with PDMS-filled hollow-core fiber, Sensors and Actuators A: Physical, 275 (2018) 60-66.
5. I. Hernandez-Romano, D. Monzon-Hernandez, C. Moreno-Hernandez, D. Moreno-Hernandez, J. Villatoro, Highly Sensitive Temperature Sensor Based on a Polymer-Coated Microfiber Interferometer, IEEE Photonics Technology Letters, 27 (2015) 2591 - 2594.
6. M. Ge, Y. Li, Y. Han, Z. Xia, Z. Guo, J. Gao, S. Qu, High-sensitivity double-parameter sensor based on the fibre-tip Fabry–Pérot interferometer, Journal of Modern Optics, 64 (2017) 596-600.
7. T. Salunkhe Tejaswi, K. lee Ho, W. Choi Hyung, J. Park Sang, H. Kim Ji, T. Kim Il, High sensitivity temperature sensor based on Fresnel reflection with thermosensitive polymer: control of morphology and coating thickness, Japanese Journal of Applied Physics, 59 (2020) SGGG06-1-SGGG06-7.
8. Salunkhe, Lee, Choi, Park, I.T. Kim, Enhancing Temperature Sensitivity of the Fabry–Perot Interferometer Sensor with Optimization of the Coating Thickness of Polystyrene, Sensors, 20 (2020) 794.
9. T. Ayupova, M. Shaimerdenova, D. Tosi, Shallow-Tapered Chirped Fiber Bragg Grating Sensors for Dual Refractive Index and Temperature Sensing, Sensors, 21 (2021) 3635.
10. X. Sun, L. Zhang, L. Zeng, Y. Hu, J.-a. Duan, Micro-bending sensing based on single-mode fiber spliced multimode fiber Bragg grating structure, Optics Communications, 505 (2022) 127513.
11. H. Niu, W. Chen, Y. Liu, X. Jin, X. Li, F. Peng, T. Geng, S. Zhang, W. Sun, Strain, bending, refractive index independent temperature sensor based on a graded index multimode fiber embedded long period fiber grating, Opt Express, 29 (2021) 22922-22930.
